# Prevalence of goiter and its associated factors among primary school children in Chole District, Arsi Zone, Ethiopia: a cross- sectional study

**DOI:** 10.1186/s40795-018-0267-2

**Published:** 2019-02-01

**Authors:** Abera Bekele, Takele Menna Adilo

**Affiliations:** 1 Assela Town Administration Health Office, P.O.Box 32, Assela, Ethiopia; 2 Department of Public Health, St Paul’s Hospital Millennium Medical College, P.O. Box, 1271 Addis Ababa, Ethiopia

**Keywords:** Factors associated, Goiter, Iodine deficiency, Primary school children, Ethiopia

## Abstract

**Background:**

Goiter remains one of the major public health problems particularly among young children in economically disadvantaged countries like Ethiopia. The aim of the study was to assess the prevalence of goiter and its associated factors among children aged 6–12 years in Chole district, Arsi Zone, Eastern Ethiopia.

**Methods:**

A school based cross-sectional study was conducted in February, 2017 among 422 primary school children in Chole district, eastern Ethiopia. The schools and study subjects were randomly selected. A structured, pretested and interviewer- administered questionnaire was used to collect the required data. It was conducted after getting due consents from the school administration and assent from caregiver/parent. Spot testing kits were used to estimate the level of iodine in salts. Descriptive statistics, cross-tabulations for chi-square test, and bivariate and multivariate logistic regression models were used to show the magnitude of goiter and its associated factors. Odds ratios with 95% confidence intervals were computed to determine the presence and strengths of associations.

**Results:**

From the 422 study participants, 407 (96.4%) completed the questionnaire. Of these 205(50.3%) were female. The mean age of participant school children was 9.87(SD ± 1.6) years. The prevalence of goiter among study subjects was 36.6% (95% CI, 31.6–40.8%). History of goiter in the family (AOR = 6.80; 95% CI: 3.34–13.84), cabbage consumption (AOR = 2.52; 95% CI: 1.38–4.60) and living with family in a single room (AOR = 2.30; 95% CI: 1.13–4.67) were positively associated with the development of goiter among primary school children in Chole district, eastern Ethiopia. But consuming milk (AOR =0.37; 95% CI: 0.23–0.59) was found to be negatively associated or protective against the development of goiter among the study subjects.

**Conclusions:**

Iodine deficiency was found to be significant public health problem in the study area. Consuming milk was found to be protective, whereas consuming goitrogenic foods like cabbage were found to be the risk factors for the development of goiter among school -aged children. Thus, ensuring the consumption of iodized salt and promoting iodine rich food items among the community in Chole district and other similar settings in Ethiopia are strongly recommended.

## Background

Goiter remains as one of the major health problems particularly among the young children and pregnant women worldwide [1]. Iodine is a crucial constituent of hormones formed by the thyroid gland and goiter is the most visible sequel of iodine deficiency in human life [2]. Globally, two billion people are at risk of Iodine deficiency disorders (IDD) due to insufficient intake of iodine and about 266 million school-aged children are at risk of insufficient iodine intake related health problems [3].

Goiter is one of the most pathological manifestations of long term depletion of iodine storage in human body especially among children living in iodine deficient areas [4]. It is caused mainly by inadequate intake of iodine containing foods and high consumption of goitrogenic foods such as cabbage, maize, sweet potato and millet. The goitrogenic foods contain thiocyanate and isothiocyanate that restrain thyroid iodide transport [4, 5]. Insufficient production of thyroid hormone in the body is also a risk factor for goiter formation, besides inadequate iodine intake [1].

Iodine deficiency during childhood would reduce cognitive, somatic growth and motor function [6, 7]. It has multiple adverse effects in humans, and these effects are best explained as iodine deficiency disorders. The most common outcome of iodine deficiency during childhood is developmental delay [8].

Despite the fact that the World Health Organization (WHO) stated goiter as the sole most preventable cause of mental retardation and brain damage its prevalence among the population of the world is still estimated to be 15.8% [4, 9]. The burden of goiter is more prevalent in low income countries like Ethiopia [2].

A complete review on Iodine deficient disorder which was conducted in 2012 reported that the Africa continent harbors the highest burden of goiter rate which was between 4.7 to 28% [4].

As a study shows about 62% of the total population of Ethiopia are at risk of Iodine deficiency disorder. In addition, an estimated 12 million school -age children in the country were exposed to in adequate iodine intake related health problems [10]. Thus, Ethiopia is considered as one of the countries in the globe with most people vulnerable to in sufficient iodine intake.

This can be explained further by the 14% increase in the prevalence rate of goiter among the total population of Ethiopia between 1980 to 2009,i.e. from 26 to 40%. The high IDD related peri- natal death of about 50,000 per year in the country is another important indication for the severity and magnitude of IDD in the country [11–13].

Furthermore, the proportion of children with goiter ranged from 15 to 30% in different regions of the country. The accessibility of iodine that is supposed to have effect on the prevalence of iodine deficiency disorder in iodine deficient areas in Ethiopia are believed to be caused by many ecological and nutritional predictors. This could be further explained by the mountainous landscape of many of the regions in the country which could result in the washing away of important nutrients like iodine because of the likely occurrences of repeated erosions in the areas for many years [14].

As a result, crop growing of the areas could be either with very low or no iodine content. For instance, as the findings of a study conducted in Bale Zone of Oromia region on iodine content of edible salt, cereals and drinking water showed there was very low amount of iodine and high concentration of other goitrogenic minerals in drinking water of the area [15].

Among the most vulnerable population group, school age children are particularly important for the assessment of IDD due to their high vulnerability. Studies reported that the prevalence of goiter among school age children of a certain area is an indicator for the status of iodine consumption in the community [16–22].

Other studies conducted previously in different countries reported that the prevalence of goiter among school children aged from 6 to 12 years varies from country to country. For instance, it was reported as 19.8% in the Karnataka, India, 35% in Pakistan and 48.3% at Kashmir Valley, in India [16, 21, 22]. As a study conducted in 2007 reported the prevalence of goiter among school children aged 6–12 years in Ethiopia was 39.9% [13].

Universal salt iodization (USI) is one of the most cost effective development attempts that can have paramount importance in improving the economic and social development of a country. As the World Health Organization (WHO) report indicated, about70% of the population of the world had access to iodized salt at household level [7, 20]. But the Ethiopian Demographic Health Survey (EDHS) of 2011 report indicated that only 15% of the households had access to an adequate amount of iodized salt in the country [23, 24].

In addition, WHO recommended that if the Total Goiter Rate (TGR) which is equivalent to the number of goiters of grades 1 and 2 detected in a population divided by the total number of individuals examined is 5% and above among children aged 6–12 years, it is considered as public health significant problem [7, 13]. Moreover, the daily recommended dietary allowance of iodine for school aged Child or 6–12 years old in order to prevent goiter is about 120 μg [25, 26].

However, most of the people in Ethiopia including our study area, Chole district live in the mountainous areas that are more vulnerable to erosions and flooding as well as subsequent risk of prevalent iodine deficiency in the community.

Cognizant of the problem, the Government of Ethiopia had launched a five –year national plan to eradicate iodine deficiency and associated health problems by the year 2015 through the achievement of utilization of adequately iodized salt to 90% of the population in the country [10, 12, 23]. In addition, the Federal Ministry of Health designed a National Nutrition Program and Micronutrient Guideline, and endorsed a proclamation for ensuring the availability of iodized salt [12, 24], though significant changes have not been attained and updated data are still scarce [10, 24, 27, 28].

Furthermore, even though many efforts were undertaken to minimize the problem, goiter is still prevalent in Ethiopia mainly among children and women living in high land areas including our study area or Chole district. Assessing the burden of ID among the most at risk population segment or school children might have paramount significance to clearly understand the progress of the current interventions and also to plan for sound actions in the future based on timely and research- based evidence.

Therefore, this study was designed to fill the current research gap about the prevalence and associated factors of goiter among school children (6–12 years) in Chole District, Oromia region, Ethiopia.

## Methods

### Study area/setting

The study was conducted in Oromia Regional State from February 5 to 25, 2017 with the aim to assess the prevalence and associated factors of goiter among primary school children aged 6–12 years.. Over 60% of the area of Chole district is highland. The estimated total population of the district for the year 2016 was 113,798, and of which 57,791 (50.8%) were female [29]. There were four health centers, 18 health posts and six different categories of private clinics, 3 Kindergartens (KGs), 44 primary and 5 secondary schools during the year 2016/17 in Chole district [30].

### Study design and population

A school based cross-sectional study was conducted among selected primary schools children in Chole district using systematic random sampling technique. Although the study focused on all the school children in the district aged 6–12 years, who were available during the study period, few students who were unable to communicate effectively because of some of the health problems of hearing and speaking during the data collection period were excluded.

### Sample size and sampling technique

The sample size of the study was calculated using a single population proportion formula. It was calculated considering the proportion (P) of goiter among school children based on the finding from the similar study done previously in Goba town, Eastern Ethiopia which was 50.0% [28]. In addition, 95% Confidence Interval (CI), 5% desired precision, and 10% contingency for the non response rate were considered during the sample size calculation. Accordingly, the calculated total sample size was 422 primary school children.

As the sampling technique was considered, firstly, the number of schools in the district was obtained from Chole district education office. Secondly, among the primary schools in the study area during the study period, four primary schools were selected using simple random sampling technique. Thirdly, the sample size was allocated to each of the four primary schools proportionately. Fourthly, grades of school children were selected using simple random sampling technique. Finally, systematic sampling technique was used to select all the targeted 422 students from four primary schools. Students who were selected but absent on the day of data collection were replaced by the next student from the same class.

### Study variables and measurement

The outcome variable of this study was goiter status and its presence or absence was assessed using inspection and palpation techniques of the physical examination [28, 30–32].

The risk factors used to determine associations with goiter among school children were some of the selected socio-demographic characteristics of the study participant children and their parents. The socio- demographic characteristics or variables were chosen based on theoretical backgrounds or findings of previous similar studies. In addition, children’s dietary or eating practices in their respective households, focusing on the recall of at least a week proceeding the data collection period were assessed and computed for their level of significance with presence or absence of goiter among the study participants.

### Data collection methods and quality control

An interviewer –administered, structured and pre-tested questionnaire was used to collect the data. Pre-testing of the questionnaire was carried out in a primary school that had similar characteristics with the schools selected for the actual data collection and then necessary corrections on the questionnaire were made accordingly. The data collection process was conducted by four senior health professionals with experiences in similar data collection procedures after being trained for two days on the concepts and contents of this study. Two experts in nutrition and the investigators of this study supervised all the process of data collection closely to maintain the required quality.

As studies indicated the prevalence of goiter among children of a certain area can be taken as an indicator for the status of iodine consumption in the community or the likelihood of iodine deficiency disorder in the area [28, 32].

Accordingly, at the day of physical examination for determining the status of goiter among schoolchildren, the participants were instructed to bring a handful of salt used by their home. The iodine content of the sample salt was tested by rapid iodized salt test kit. The salt sample was taken in a teaspoon and then a drop of the test solution was poured on the salt. Salt samples with less than 15 ppm of iodine content were classified as inadequate iodine [10, 28].

Since 1960, inspection and palpation methods have been used for assessment of the severity of endemic goitre in populations [2]. Accordingly, in this study the physical examination of children were done by experienced two health officers using inspection and palpation techniques to determine thyroid size or goiter. The goiter was graded when a student was in a normal position as 0, 1 and 2. Consequently, Grade 0 (neither palpable nor visible goiter), grade 1 (palpable goiter but not visible) and grade 2 (a swelling in neck that is clearly visible). Then, the total goiter rate (TGR) was calculated by adding grade 1 and 2 and dividing to the total number of children examined to express in percentages ([17, 19, 21, 22] 281).

Furthermore, the collected data on socio-demographic characteristics and dietary practices of school children and their respective parents as well as the status of goiter among school children were checked first for their completeness and then cleaned and checked for accuracy, consistencies and missed values before conducting analysis.

### Statistical analysis of the data

The data analysis was done using SPSS computer software version 20.0. Descriptive statistics like frequency distribution and percentage were employed using tables and figures. Bivariate and multivariate logistic regression models were performed to identify factors associated with goiter among primary school children in study area.

Variables that have shown statistically significant associations in bivariate analyses or *P*- value less than 0.05 as well as those variables with the theoretical associations were entered in to multiple logistic regression models to assess for their independent contributions. The variables with theoretical associations refer to the variables age, consuming maize, and iodine content of the salt that didn’t show statistically significant associations with the status of goiter among school children during bivariate analysis of this study despite the fact that they were reported as risk factors for the development of goiter among school –aged children in some previous similar studies [5, 33–35]. The presence of association was determined by the odds ratio and level of significance (*P*-value < 0.05).

## Results

### Socio- demographics characteristics of the study participant children

From the total 422 study participants 407(96.6%) completed the questionnaire. All the students who completed the questionnaire were involved in the analysis. The mean age of the study subjects was 9.87 (+ 1.6) years. Among the study participants 205 (50.4%) were female, 216(53.1%) were Oromo and the rest 191 (46.9%) were Amhara by ethnicity. The majority of the study subjects 263(64.6%) were Orthodox Christians Table 1.

**Table 1:** Scio-demographic characteristics and prevalence of Goiter among the study participants (Primary school Students) in Chole district, Oromia region, Ethiopia; 2017

Variables		Frequency	Percentage
Sex	Male	202	49.6
	Female	205	50.4
Ethnicity	Oromo	216	53.1
	Amhara	191	46.9
Religion	Orthodox	263	64.6
	Muslim	138	33.9
	Protestant	6	1.5
Education status of fathers	illiterateUn educated	89	21.9
	Primary school (grade 1–8)	269	66.1
	Secondary school and above	49	12
Education status of mothers	Illiterate/Un educated	218	53.6
	Primary (1–8)	169	41.5
	Secondary (9–12) and above	20	4.9
Occupation of fathers	Farmer	389	95.6
	Government employees	9	2.2
	others	9	2.2
Occupation of mothers	House wife	401	98.5
	Others	6	1.5
Prevalence of Goiter among primary school children	Male	71	35.1
	Female	78	38.0
	Total	149	36.6

### Dietary factors associated with goiter among school children

Goitrogens foods were frequently eaten by children. From the total 407 children included in the study, 322 (79.1%) were consuming cabbage, 327(80.3%) were consuming maize, 147(36.1%) were consuming millet and 76(18.7%) were consuming sweet potato at least once during the previous week. In addition, of the study participants 269(66.1%) were consuming milk which is non goitrogenic at least once during the previous week.

Of all the 407 study subjects who were asked to bring the sample of salts from what their respective family is consuming during the data collection period, 387(95.1%), 8(2.0%) and 12 (2.9%) brought salts with no iodine (ppm = 0), inadequate iodine content (ppm < 15 and sufficient amounts of iodine (ppm > 15, respectively (Table 2).

**Table 2:** Dietary factors and availability of iodized salt among primary school students in Chole district, Arsi Zone, Oromia; 2017

Variables		Frequency	Percentage
Consumed cabbage during the past week	Yes	322	79.1
	No	85	20.9
Consumed maize during the past week	Yes	327	80.3
	No	80	19.7
Consumed Sweet potato during the past week	Yes	76	18.7
	No	331	81.3
Consumed Millet during the past week	Yes	147	36.1
	No	260	63.9
Consumed milk during the past week	Yes	269	66.1
	No	138	33.9
Iodine content of house hold salt	>15PPM	12	2.9
	<15PPM	8	2
	0 PPM	387	95.15

### Prevalence of goiter among the study participants

The observed prevalence of goiter among study participants was 36.6%. Of these 29% were with grade one (palpable) goiter and 7.6% were with grade two (visible) goiter. The prevalence of goiter was higher among female students than males. It was 38.0 and 35.1% among females and males, respectively.

### Factors associated with goiter in bivariate analysis

In bivariate analysis, history of goiter in the family (COR = 6.68;95 CI: 3.43–13.01), living with family in a single room (COR = 2.21; 95 CI: 1.15–4.27), consuming cabbage (COR = 2.17; 95 CI: 1.26–3.76) and consuming milk (COR = 0.40, 95% CI: 0.26–0.62) were found to be factors associated with development of goiter among school children in Chole district (Table 3, Annexed on Page 23).

**Table 3:** Bivariate Logistic regression analysis of factors associated with goiter among primary school Students aged 6–12 in Chole District, Arsi Zone, Oromia Regional State, Ethiopia, 2017

Variables	Goiter Status of Student		COR (95% CI)
	Yes (%)	No (%)	
Socio-economic and demographic Factors			
Age			
6–8 years	29 (30.5)	66 (69.5)	1.0
9-12 years	192 (61.5)	120 (38.5)	1.422 (0.87–2.33)
Sex			
Male	71 (35.1)	131 (64.9)	1.0
Female	78 (38.0)	127 (62.0)	1.13 (0.75–1.7)
Student’s Father Education Level			
Illiterate/Un educated	32 (36.0)	57 (64.0)	1.1 (0.51–2.2)
Primary school(1–8)	100 (37.2)	169 (62.8)	1.1 (0.59–2.1)
Secondary school and above	17 (34.7)	32 (65.3)	1.0
Student’s Mother Education Level			
Illiterate/Un educated	83 (38.1)	135 (61.9)	1.14 (0.44–2.98)
Primary school (1–8)	59 (34.9)	110 (65.1	1.0 (0.38–2.63)
Secondary school and above	7 (35.0)	13((65.0)	1.0
Number of rooms in a house			
One	43 (75.4)	14((24.6)	2.21 (1.15–4.27)
Two	132 (58.0)	95 (42.0)	1.49(.94–2.37)
Three and above	40 (33.0)	83 (67.0)	1.0
Family Size			
2–4	42 (31.9)	90 (68.2)	1.0
5–7	168 (61.0)	107 (39.0)	0.73 (0.47–1.14)
History of goiter in the family (/They were asked if they had heard the history of swellings on the theneck			
Yes	39 (75.0)	13 (25.0)	6.68 (3.43–13.01)
No	110 (31.0)	245 (69.0)	1.0
Dietary factors			
Consumed Cabbage during last week			
Yes	193 (60.0)	129 (40.0)	2.17 (1.26–3.76)
No	20 (23.5)	65 (76.5)	1.0
Consuming maize during last week			
Yes	127 (38.9)	200 (61.1)	1.67 (0.97–2.87
No	22 (27.5)	58 (72.5)	1.0
Consuming Sweet Potato during last week			
Yes	28 (37.3)	47 (62.7)	0.96 (0.57–1.62)
No	121 (36.4)	211 (63.6)	1.0
Consuming Millet during last week			
Yes	56 (38.1)	91 (61.9)	1.11 (0.73–1.67
No	93 (35.8)	167 (64.2)	1.0
Consuming milk during last week			
Yes	79 (29.4)	190 (70.6)	0.40 (0.26–0.62)
No	70 (50.75)	68 (49.3)	1.0
Iodine content of the salts			
PPM = 0	250 (64.6)	137 (35.4)	3.04 (0.72–12.92
PPM < 15	5 (62.5)	3 (37.7)	2.56 (0.80–8.20)
PPM > 15	7 (58.3)	5 (41.7)	1.00

### Factors associated with goiter in multivariable analysis

The variables history of goiter in the family (AOR =6.80;, 95% CI: 3.34–13.84), living together with parents in a single room (AOR = 2.30; 95% CI: 1.13–4.67), consuming cabbage (AOR = 2.52; 95% CI: 1.38–4.60) and, consuming milk (AOR = 0.37; 95% CI: 0.23–0.59) at least once in a week or more frequently had shown statistically significant associations with the outcome variable or occurrences of goiter among school children by controlling for potential confounders (Table 4, Annexed on page 25).

**Table 4:** Multivariable Logistic regression Analysis of Factors Associated with Goiter among primary School Students aged 6–12 years in Chole District, Arsi Zone, Oromia Regional state, Ethiopia, 2017

Variables	Goiter Status of Student		COR(95%, CI)	
	Yes (%)	No (%)		AOR(95 %CI)
Age				
6–8 years	29 (30.5)	66 (69.5)	1.42 (0.87–2.33)	1.45 (0.83–2.56)
9-12 years	192 (61.5)	120 (38.5)	1.0	1.0
Number of rooms in parents house				
One	43 (75.4)	14((24.6)	4.21 (1.15–4.27)	2.30 (1.13–4.67)
Two	132 (58.0)	95 (42.0)	1.49(.94–2.37)	1.51 (0.67–3.25)
Three and above	40 (33.0)	83 (67.0)	1.0	1.0
Family Size				
2–4	42 (31.9)	90 (68.2)	1.0	0.74 (0.45–1.21)
5–7	168 (61.0)	107 (39.0)	0.73 (0.47–1.14)	1.0
Consuming Cabbage during last week				
Yes	193 (60.0)	129 (40.0)	2.17 (1.26–3.76)	2.52 (1.38–4.60)
No	20 (23.5)	65 (76.5)	1.0	1.0
Consuming milk during last week				
Yes	79 (29.4)	190 (70.6)	0.40 (0.26–0.62)	0.37 (0.23–059)
No	70 (50.75)	68 (49.3)	1.0	1.0
History of goiter in the family/ was asked in local language /understandable way				
Yes	39 (75)	13 (25.0)	6.68 (3.43–13.02)	6.80 (3.34–13.84) 1.0
No	110 (31.0)	245 (69.0)	1.0	
Consuming Maize during last week				
Yes	127 (38.9)	200 (61.1)	1.0	
No	22 (27.5)	58 (72.5)	1.67 (0.98–2.87)	1.78 (0.97–3.27) 1.0
Iodine content of the salt				
PPM = 0	250 (64.6)	137 (35.4)	3.04 (0.72–12.92	3.08 (0.64–14.91)
PPM < 15	5 (62.5)	3 (37.7)	2.56 (0.80–8.20)	2.95 (0.82–10.69)
PPM > 15	7 (58.3)	5 (41.7)	1.0	1.0

## Discussion

As the findings of this study showed, the prevalence of goiter among primary school students aged 6–12 years in the study area was 36.6%, and it was in line with the national prevalence (39.9%), and that of the finding of a study conducted among children aged 6–12 years in rural northwest Ethiopia (37.6%) [5, 10, 12]. However, this prevalence was lower than the finding of a study conducted in Shebbe Sonbo District, Jimma Zone with higher altitude and rain fall because of its topography [31]. The difference could be explained in terms of variations in altitude and annual rain fall between the two study areas and the subsequent likely erosions of the top soil rich with iodine content [31]. But the observed prevalence of goiter among the participant school children of this study was higher than the prevalence of goiter among school –aged children reported in studies conducted in India [21, 22]. The possible explanations for higher prevalence of goiter among children in our study comparing with the findings of other similar studies in Asia (Karnataka, Kashmir Valley and Pakstan) could be due to the mountainous nature of Ethiopia and poor soil conservation for long period of time which might have contributed to the removal of iodine rich top layer of the soil [12, 13, 16, 21, 22, 31].

The findings of this study also revealed that, the prevalence of goiter was higher in the older group of children. It was also in agreement with the findings of a study that was done in Goba town where the prevalence of goiter among age group 6–8 was reported 39% and among those aged 9–12 it was 53.7% [36]. The increase in prevalence of goiter with age may be because of the amount of iodine required by body as the age increases [6].

In addition, our findings indicated that the prevalence of goiter was higher among females than males. Higher prevalence of goiter among females may be due to the fact that iodine requirement for female children are higher than males especially at the beginning of pubertal age [32]. The females are more vulnerable to goiter due to physiological reasons like early puberty, which starts about 2 years earlier than males. Nonetheless, sex as a variable didn’t show statistical significance with the status of goiter in bivariate analysis which concurs with the findings of a similar study that was conducted among children in Wolaita, Ethiopia [32].

School -aged children who reported family history of goiter were about six times more likely to have goiter compared to their counterparts. This finding is in agreement with the previous study that was done in North West Ethiopia [5]. The consistency in findings regarding the associations between family history and goiter among school children in Ethiopia could be explained in terms of a number of environmental and dietary factors associated with iodine deficiency among different segments of population that are inter-generational in many parts of the country due to either poverty or low coverage of national salt iodization program [11–15]. Children who lived in a single room together with their parents were more likely to develop goiter when compared with those who lived in a house with two or more rooms. This finding is also consistent with the finding of a study conducted in Goba town [28]. It could be explained in terms of the variation in nutritional status or quality of daitry intake of a community or a family‘s iodine intake [28].

Children who consumed cabbage at least once in a week or more frequently were more likely to develop goiter than those who had not. It was also consistent with findings of similar other studies that were conducted previously in different parts of the country i.e. in Goba Town, in Shebbe Senbo district, Jimma zone and Wolayita Soddo Town [28, 31, 32]. The possible explanation for this could be due to cabbage being a natural goitrogens food that contains thiocyanate and isothiocyanate, which inhibit thyroid iodide transport. The inhibited thyroid iodide transport in our body, may result in the enlargement of thyroid gland [32]. Thus, continuous efforts through health education should be done to ensure the consumption of iodized salt among the residents of Chole district and other similar settings through increased awareness of community on the benefits of iodine rich diets such as cow milk,butter and cheese. In addition, more efforts to make the iodized salt accessible to the community can have paramount importance as a solution.

Those study participant children who were consuming milk at least once in a week were less likely to develop goiter comparing with their counterparts This finding also concords with the reports from different sources that indicated cow milk contains useful nutrient for prevention of goiter [37, 38]. Even though foods from animal origin have less amount of iodine than the foods from plant origin, milk and dairy are fairly good source of iodine [37, 38].

Furthermore, as salt is frequently consumed by everyone, consumption of iodized salt is the easiest and cheapest modality of IDD prevention and control. The Federal Democratic Republic of Ethiopia (FDRE) passed a mandatory salt regulation and monitoring requiring all salt meant for human consumption to be iodized since March 2011 [36, 39].

However, our study findings indicated that only 2.9% of the families of school children who participated in this study had sufficient levels of iodine in their salt (> 15 ppm). The finding of our study was lower than the findings of Ethiopian Demographic and Health Survey or EDHS 2011 which reported 15% of the households’ access iodized salt [23]. The accessibility of sufficient (15 ppm) iodized salt in this study was also lower than the study conducted in Northwest Ethiopia and Goba town which were 29 and 29.9%, respectively [10, 28]. This could be explained in terms of low coverage of iodization of salt to the community which might be improved through an efficient universal salt iodization national program with due emphasis to the most iodine deficient districts like Chole, our study area [23, 24, 36, 39].,

Furthermore, as recommended by the World Health organization(WHO), 90% of the households in a population should have access to use salt that contains the iodine level of > 15 ppm for the effective elimination of iodine deficiency disorder or goiter [17, 19]. Additionally, the proportion of people who are consuming food items that contain adequate iodine such as cow milk in our study area was found to be very low. This could be explained in terms of low income among many families in the study area which could also have resulted in the observed high prevalence of goiter among the study participant school children.

Thus, periodic assessment of locally available food items with fairly high amount of iodine and the content of house hold salts for ensuring the recommended content of iodized salt to the community can have paramount importance in decreasing the prevalence of goiter and its effects among school children.

## Limitation

In this study urinary iodine level of the study subjects that could reveal recent iodine intake status and could also help for treatment and monitoring was not tested due to resource constraints. Since the study was conducted in the school, the storage of the salt in the home wasn’t observed which might also contribute as factor of goiter.

In addition, as this study was a cross –sectional study design, determining the causality of goiter is not strong. Furthermore, some of the information like family history of goiter that was collected from school children might lack accuracy and need to be cross checked with the information from the students’ respective parents.

## Conclusions

The study revealed that primary school children in the study area were severely affected by iodine deficiency, and the area can be classified as goiter endemic. Family history of having goiter, consuming cabbage, living together with family in a single room and inability to consume milk at least once in a week were found to be the likely risk factors for having goiter among school children. Supplying iodized oil capsule to school children, promoting iodine rich foods and ensuring iodized salt through effective universal salt iodization program in the community are believed to play significant role in decreasing the prevalence of goiter and its associated health effects among primary school children. Finally, periodic assessment of iodine content in house hold salts for ensuring the recommended content of iodized salt to the community can also have paramount importance in decreasing the prevalence of goiter and its effects among school children in Chole district and other similar settings in low income countries including Ethiopia.

## Abbreviations

AOR: Adjusted Odds Ratio
CI: Confidence Interval
COR: Cumulative Odds Ratio
EDHS: Ethiopian Demographic and Health Survey
FDRE: Federal Democratic Republic of Ethiopia
IDDs: Iodine Deficiency Disorders
IRB: Institutional Review Board
KGs: Kindergartens
PPM: Parts Per Million
SD: Standard Deviation
SPHMMC: St Paul’s Hospital Millennium Medical College
SPSS: Statistical Package for Social Sciences
TGR: Total Goiter Rate
USI: Universal Salt Iodization
WHO: World Health Organization
